# Predictors of Mental Health During the COVID Pandemic

**DOI:** 10.1093/geroni/igab046.1581

**Published:** 2021-12-17

**Authors:** Helen Lach, Devita Stallings, Rebecca Lorenz, John Taylor, Janice Palmer

**Affiliations:** 1 Saint Louis University, Saint Louis, Missouri, United States; 2 Saint Louis University, St. Louis, Missouri, United States; 3 University at Buffalo, Buffalo, New York, United States; 4 Saint Louis University, Hillsboro, Missouri, United States; 5 Webster University, St. Louis, Missouri, United States

## Abstract

Health professionals have been concerned about mental health of older adults during the COVID pandemic. To explore their experiences, we conducted an online survey of community-dwelling older people to examine their mental health related to stress, based on Pearlin’s Stress Process Model. A snowball approach was used; we sent recruitment e-mails through senior organizations and contacts with e-mail lists of potential participants; there were 504 respondents. We used regression analysis to explore predictors of mental health based on Pearlin’s model. Background characteristics included age (m = 75.7, SD 4.95), gender (77.4% female) and race (White = 93.4%). The CESD-10 provided a measure of mental health. Scores indicated 62.3% of the sample scored in the low range for depressive symptoms and 37.7% in the moderate to high range. Stressors were measured using the Perceived Stress Scale that includes subscales of perceived helplessness and perceived self-efficacy. We also measured perceived social Isolation, and current life space as predictor variables. Results of regressing the CESD-10 onto the set of theoretical predictors revealed that the inclusion both subscales of the Perceived Stress Scale, social isolation, and current life space jointly accounted for approximately 63.0% of the variability in the outcome beyond the baseline model (FChange[4, 449] = 211.15, p < .01), which included age, race, and gender. The model overall, accounted for approximately 66.5% (R2adjusted = 66.0%) of the variability in CESD-10 scores, (F[7, 449] = 127.473, p < .01). Addressing stress among older adults is important to help them maintain positive mental health.

